# Histamine Intolerance—The More We Know the Less We Know. A Review

**DOI:** 10.3390/nu13072228

**Published:** 2021-06-29

**Authors:** Martin Hrubisko, Radoslav Danis, Martin Huorka, Martin Wawruch

**Affiliations:** 1 Department of Clinical Allergology and Immunology, Oncological Institute of St. Elizabeth, Heydukova 2157/10, 812 50 Bratislava, Slovakia; martin.hrubisko@ousa.sk; 2 Institute of Immunology and Allergology, Slovak Medical University, Limbová 12, 833 03 Bratislava, Slovakia; 3 Institute of Pharmacology and Clinical Pharmacology, Faculty of Medicine at Comenius University of Bratislava, Špitálska 24, 831 72 Bratislava, Slovakia; martin.wawruch@fmed.uniba.sk; 4 Department of Gastroenterology and Hepatology, University Hospital Bratislava, Ružinovská 6, 821 01 Bratislava, Slovakia; martin@huorka.sk

**Keywords:** histamine intolerance, histamine, diamine oxidase, DAO, low-histamine diet, probiotics, food intolerance

## Abstract

The intake of food may be an initiator of adverse reactions. Food intolerance is an abnormal non-immunological response of the organism to the ingestion of food or its components in a dosage normally tolerated. Despite the fact that food intolerance is spread throughout the world, its diagnosing is still difficult. Histamine intolerance (HIT) is the term for that type of food intolerance which includes a set of undesirable reactions as a result of accumulated or ingested histamine. Manifestations may be caused by various pathophysiological mechanisms or a combination of them. The problem with a “diagnosis” of HIT is precisely the inconstancy and variety of the manifestations in the same individual following similar stimuli. The diagnosing of HIT therefore requires a complex time-demanding multidisciplinary approach, including the systematic elimination of disorders with a similar manifestation of symptoms. Among therapeutic approaches, the gold standard is a low-histamine diet. A good response to such a diet is considered to be confirmation of HIT. Alongside the dietary measures, DAO supplementation supporting the degradation of ingested histamine may be considered as subsidiary treatment for individuals with intestinal DAO deficiency. If antihistamines are indicated, the treatment should be conscious and time-limited, while 2nd or 3rd generation of H_1_ antihistamines should take precedence.

## 1. Introduction

The intake of food may be an initiator of adverse reactions. We are referring to any kind of abnormal reaction related to the intake of foods. Adverse food reactions are nowadays rather accepted in practice but are however less frequently objectively examined [1]. In addition to specific and well-differentiated disorders, allergic reactions and food aversions, we also class food intolerance among them. Distinguishing food intolerance from an allergic reaction to food is possible on the basis of key pathophysiological differences through the use of relevant diagnostic approaches. Food allergy is an inadequate response of the immune system to an antigen (in the majority of cases of a protein nature) ingested in food, that is accompanied by IgE or non-IgE (cellular) immunological mechanisms. Its cumulative prevalence is 3–6%, appearing much more frequently in children [2]. Testing for foods IgG or IgA antibodies is not of fundamental clinical importance [3,4]. Levels of these antibodies may rather reflect an intestinal permeability disorder regardless of its origin, which is often post-infectious [5]. One specific form of an adverse food reaction with an immunopathological feature is celiac disease, in which genetic disposition and epigenetic influences lead to adverse immunity reactions to gluten.

Food intolerance is an abnormal non-immunological response of the organism to the ingestion of food or its components in a dosage normally tolerated [6]. It is at the same time a simplified term for non-allergic food hypersensitivity according to the World Health Organization (WHO) [7]. Food hypersensitivity belongs among the most frequently occurring undesirable reactions to food. It affects from 15–20% of the population and can be the result of the pharmacological effects of food ingredients, non-celiac gluten sensitivity or malfunction of enzyme(s) or transport [8].

Despite the fact that food intolerance is spread throughout the world, its diagnosing is difficult and demanding. For example, in patients with suspected histamine intolerance (HIT) it is necessary to carefully consider other possible reasons for the manifestations of symptoms (Table 1) [1]. Food intolerance (and especially HIT) requires a comprehensive understanding of the symptoms, especially their diversity, severity and time of onset [6].

In this review, we provide a critical overview on possible benefits of published diagnostic approaches. We present the current knowledge of the therapeutic options and suggest the management of HIT accordingly. Another strength of this review lies in its comprehensiveness of tables summarizing data of clinical importance.

### 1.1. Histamine Intolerance (HIT)

HIT is the term for that type of food intolerance which includes a set of undesirable reactions as a result of accumulated or ingested histamine. In the German guideline from 2017, German and Swiss specialists prefer the term “*adverse reactions to ingested histamine*” [1]. In older publications, this type of intolerance is designated by the expressions *pseudoallergy*, *enteral histaminosis* or *histamine sensitivity*. HIT is defined as a condition caused by an imbalance between the histamine released from food and the ability of the organism to degrade such an amount. It is accompanied by decreased activity of the DAO enzyme, leading to an increased concentration of histamine in plasma and the emergence of adverse reactions. In some publications, the state of decreased activity of DAO is referred to as a DAO deficiency. DAO deficiency predisposes a certain subgroup of the population to HIT. It can be of genetic, pathologic or pharmacological origin [9].

It is necessary to distinguish HIT from histamine intoxication designated as *scrombroid syndrome*, *scombroidosis* or *histamine poisoning*. The term originates from the name of the mackerel fish family (*Scombridae*), after the consumption of which the intoxication was most often observed. The *Scombridae* family includes tuna, herring and mackerel. Histamine poisoning is considered worldwide as one of the most frequent intoxications caused by the consuming of fish (Dalgaard, 2008 in [10]). According to Colombo et al., from 103 analysed samples which caused histamine poisoning, 101 showed fish or seafood sources, and only two contained cheese [10]. Manifestations of histamine intoxication may include rash, abdominal pain, vomiting, diarrhoea and shortness of breath, and the intoxication may also have a fatal outcome [11].

The term HIT is used in a similar manner as the concept of lactose intolerance (which occurs due to a lack of the lactase enzyme), since it is presumed that the HIT symptoms are related to a lack or diminished activity of the enzyme DAO. Ingested exogenous histamine is distributed into the blood stream and may trigger symptoms in the susceptible population. It should be stated that with HIT, the amount of histamine taken in is much lower than with histamine poisoning. HIT manifestations also have a milder course in comparison with intoxication [10].

With intolerances, gender-specific variations generally apply; women are affected with intolerances more frequently than men, although this distinction is not satisfactorily explained [12]. Increased sensitivity to the intake of histamine was observed in women in the premenstrual phase [13]. Serum diamine oxidase (DAO) levels in premenopausal women appear to be associated with the menstrual cycle, with higher DAO activity measured during the luteal phase compared to the follicular phase [14]. Painful menstruation may be associated with increased sensitivity to histamine. Administration of H_1_ antihistamines on the first day of menstruation has had a preventive effect on dysmenorrhea. High levels of histamine metabolites in urine during the ovulatory phase could be related to the effect of oestrogens (especially oestradiol) [15].

#### Manifestations of HIT

One of the reasons why the adverse reactions caused by the intake of histamine cannot be clearly defined and outlined, as against other sicknesses, is their heterogeneity. Due to the fact that histamine enters into the circulation and that histamine receptors occur ubiquitously in the human body, a typical clinical picture cannot be strictly defined. The adverse manifestations related to the intake of histamine are usually complex and may affect different organ systems. Paradoxically, if the set of manifestations appears in various ways, unexpectedly and randomly, and at the same time following the ingestion of food, the symptoms may have their origin in histamine intake.

**Figure 1:**
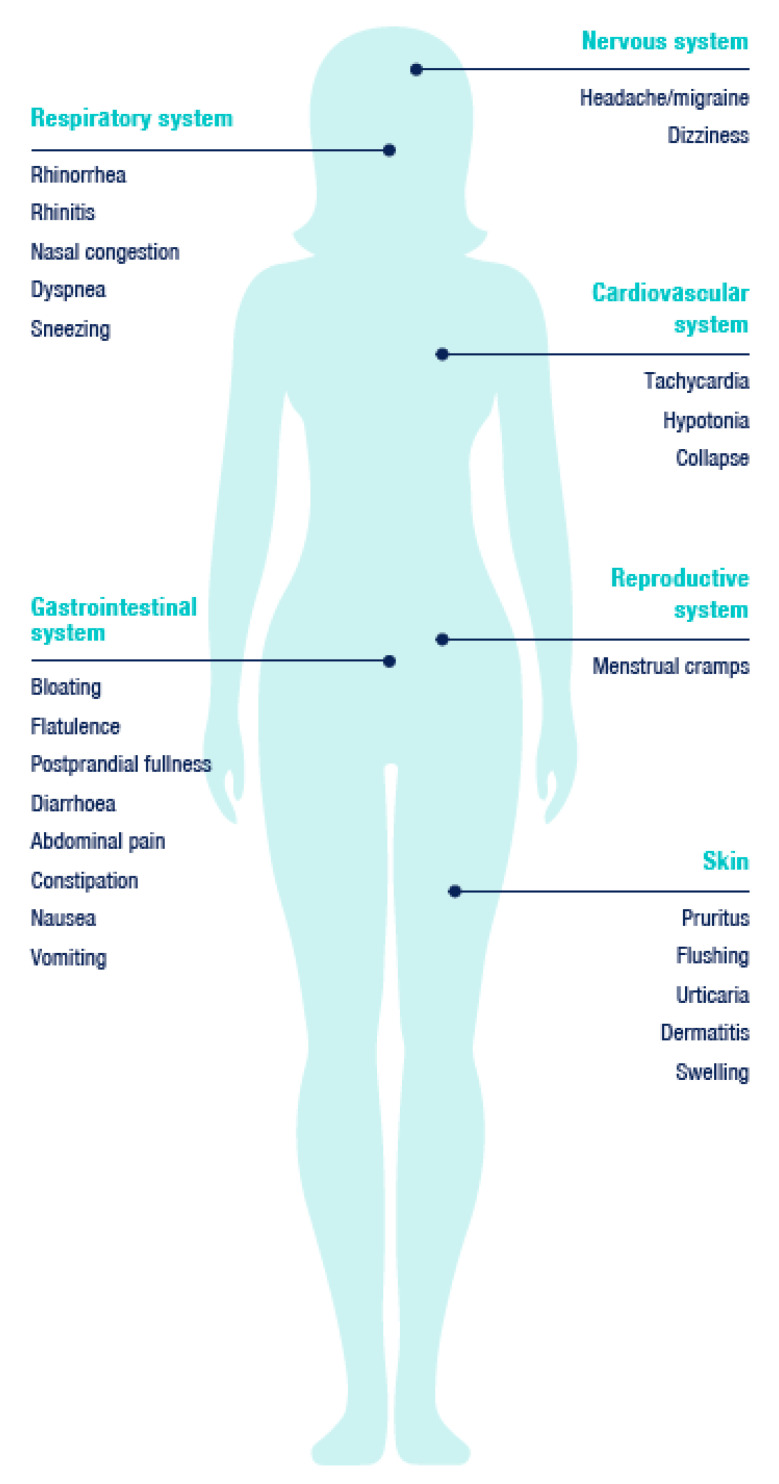
Typical manifestations related to the intake of exogenous histamine. Adapted according to [9].

As typical signs, we can observe skin manifestations—for example erythema in the facial area (flushing), pruritus, urticarial rash on the body. Gastrointestinal symptoms include diarrhoea (±vomiting) but also constipation and abdominal pain. Manifestations in the cardiovascular system, such as low blood pressure (counter-regulatory hypertension may subsequently occur) and tachycardia are less frequent [1], as are manifestations in the nervous and respiratory systems (Figure 1) [9].

The problem with a “diagnosis” of HIT is precisely the inconstancy and variety of the manifestations in the same individual following similar stimuli. In cross-over placebo-controlled trials in which symptoms were assessed, the subjects reacted randomly to the histamine provocation test. Although the total score of symptoms when histamine was administered was significantly higher as compared to the placebo, with many individuals no relationship between the ingestion of the histamine and the individual symptoms could be established [16].

New findings have been recorded just recently. A 45-year-old woman who experienced Nissen’s fundoplication for long-lasting laryngopharyngeal reflux developed episodes of throat clearing and coughing. Laryngopharyngeal reflux indicates the return flow of gastric contents to the laryngopharynx and upper aerodigestive space. It is a clinical unit different from the gastroesophageal reflux disease. In this case, consultations with nutrition specialists led to considerations of HIT. A low-histamine diet led to a significant improvement in the patient’s symptoms. For patients who do not respond according to expectations to typical laryngopharyngeal reflux treatment, a potential link to HIT should be taken into consideration [17], with a 3-month diet treatment prior to a possible operation.

In another study, 30 laryngopharyngeal reflux patients with chronic coughs underwent a histamine provocation test. Using a visual analogue scale, videolaryngostroboscopy findings and voice and throat symptoms were assessed directly before and after the exposure test. Moreover, the correlation between the relative changes in spirometry values in relation to changes in vocal fold oedema was also evaluated, along with redness and changes in the voice and throat symptoms reported by the patients during the histamine provocation test. The relative changes in inspiratory and expiratory air flow and voice and throat symptoms during the histamine challenge test correlated. Histamine induced oedema of the vocal folds, visible by videolaryngostroboscopic imaging, did not significantly influence spirometric air flow values [18].

### 1.2. Histamine

Histamine is a neuro-immuno-endocrine system mediator. In the human organism it influences the whole spectrum of physiologic functions of various tissues and cells, including immunity. From a chemical perspective, it is a ubiquitously occurring biogenic amine. In the organism, its synthesis is ensured by decarboxylation of the amino acid L-histidine by the L-histidine decarboxylase enzyme. In the human organism, histamine is primarily stored in the mast cells and basophils, but its presence has also been found in the enterochromaffin cells [19] and in the histaminergic neurons [20]. Histamine acts in the organism as an agonist of histamine H_1_, H_2_, H_3_ and H_4_ receptors. H_1_ and H_2_ receptors appear ubiquitously, with H_2_ mostly present in the digestive tract (stomach, duodenum, small intestine). The H_3_ receptors are abundant in the nervous system. The H_4_ receptors are present in certain tissues (skin, tonsils), but in a small amount [21]. Among other processes, histamine mediates inflammatory responses, vasodilation, gastric acid production in enterochromaffin cells, congestion and bronchospasm, and secretion in the respiratory system. Its pleiotropic effect was found in the nervous system, where it acts as a neuromediator and a neurohormone, influencing e.g., thermoregulation, alertness, appetite and cognitive and behavioural functions [22]. The microbiome can also be a source of histamine in the macroorganism [9]. Its production has been described in some species (see the Microbiome and HIT section). Food is the main exogenous source of histamine [23].

#### Metabolism of Histamine

The quantity of endogenous histamine is controlled on a genetic level. In genes encoding the enzymes responsible for the synthesis and degradation of histamine, similarly as in histamine receptor-encoding genes, genetic polymorphisms have been identified [21]. Genetic polymorphisms for histamine receptors and for DAO are most likely associated with several specific symptoms and their combinations [24]. In certain polymorphisms of the gene encoding DAO (and similarly, the H_3_ receptor), diminished activity of this enzyme has been reported, which increases the risk of migraines. Reduced DAO activity however has also been recorded in healthy individuals. In addition to genetic predisposition, several factors (e.g., variability of histamine content in food etc.) appear to be responsible for the manifestation of symptoms; hence the functional and clinical significance of genetic polymorphisms remains elusive [21,24].

The half-life of histamine in plasma is relatively short, a few minutes [25]. Histamine is metabolized in several pathways in the organism. As clinically most significant is considered enzymatic degradation mediated by the DAO enzyme, with a second pathway represented by the histamine-N-methyl transferase enzyme (HNMT) [1].

**Figure 2:**
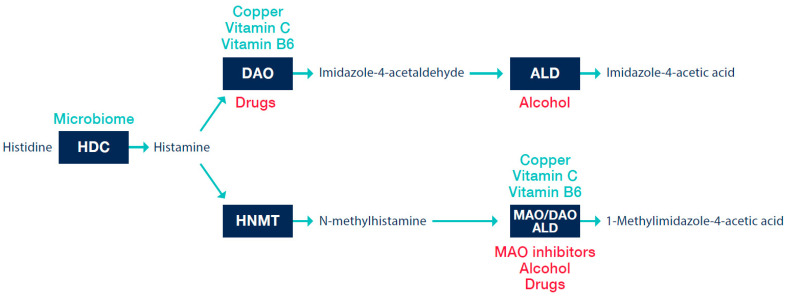
Histamine metabolism in vivo. HDC—histidine decarboxylase; DAO—diamine oxidase; ALD—aldehyde dehydrogenase; HNMT—histamine-N-methyl transferase; MAO—monoamine oxidase. The green are factors potentiating an endogenous capacity of enzymatic reaction. The red are factors directly/indirectly inhibiting an enzymatic reaction. Adapted according to [9,13,21,27].

The DAO enzyme is also identified in the literature according to the gene that encodes it, AOC1 Amine Oxidase Copper Containing 1 [26], formerly known as histaminase. The DAO molecule contains copper and is the essential enzyme responsible for the degradation of histamine from the extracellular space [27]. The product of oxidative deamination of histamine is imidazole-4-acetaldehyde (Figure 2).

**Figure 3:**
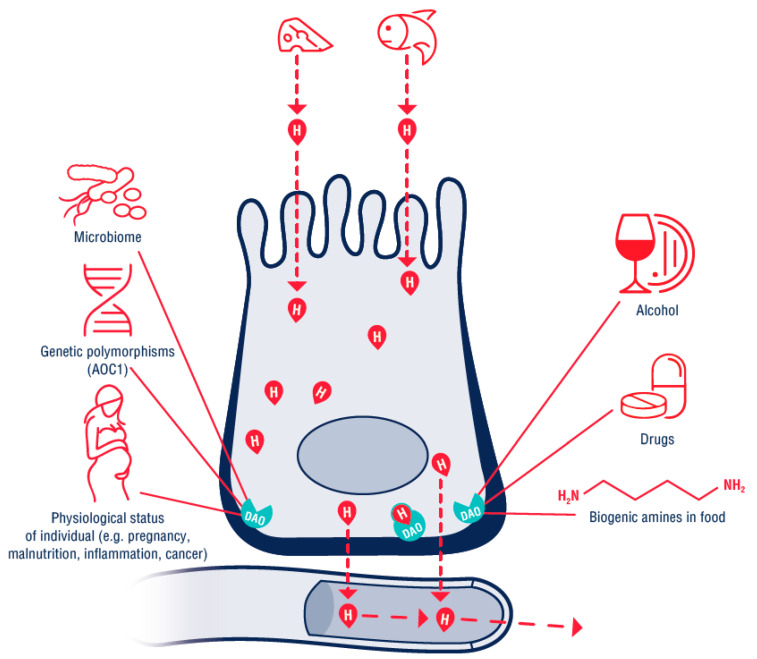
The function of diamine oxidase (DAO) in the enterocyte. The red drop with H shows the histamine released from food. The histamine passes through the enterocyte into the circulation. The DAO enzyme on the basolateral membrane creates a barrier, and the histamine obtained from the food is metabolized (the red drop with H with a green contour). DAO activity is directly/indirectly dependent on internal and external factors such as the polymorphisms of the AOC1 gene, the physiological status of the organism, alcohol, other biogenic amines and medication intake. AOC1—amine oxidase copper containing 1 gene.

DAO is found in the epithelial cells of the (small) intestine, the placenta, the kidneys, the thymus and seminal plasma [28]. The physiological function of the DAO enzyme includes regulation of the inflammation processes, proliferation, allergic response and ischemia [29]. During digestion, the DAO enzyme is continuously synthesized in the mucosa of the small intestine. It is stored in vesicular structures on the basolateral membrane of the enterocytes and acts as a metabolic barrier against exogenous diamines, including histamine [30]. The accumulation of ingested histamine and its subsequent penetration into the circulation as a result of reduced or slowed catabolism by the DAO enzyme at the level of the small intestinal epithelium is considered as a possible reason for the HIT syndrome [6]. The activity and plasmatic level of the DAO may be dependent on the genetic variability of the relevant genes (AOC1 on the 7th chromosome) [21], or on the physiological state of the organism [13]. During pregnancy, greatly increased concentrations (up to 150-fold) of serum and plasma DAO were measured [27]—the placenta is a producer of this enzyme. This is regarded as the reason why during pregnancy, in women suffering from HIT manifestations, a lessening or complete regression of HIT symptoms is observed. DAO activity or histamine release may be influenced by a number of commonly used medicaments, such as N-acetylcysteine, ambroxol, verapamil, propafenone, amiloride, cefuroxime, clavulanic acid or non-steroidal anti-inflammatory drugs, metamizole, as well as radiological contrast agents (Figure 3) [13,31]. We summarize an extended list of substances possibly interfering with the activity of DAO in Table 2.

HNMT is a cytosolic enzyme whose role is to regulate intracellular histamine levels [6]. The inactivation of intracellular histamine is mediated by the methylation of the imidazole nucleus; this metabolite is subsequently oxidized [13]. Although it is also found in the gastrointestinal tract, it is unlikely to play a major role in the degradation of exogenous histamine or histamine produced by the gut microbiome [9].

### 1.3. Biogenic Amines in Food

Biogenic amines may be present in greater or lesser amounts in any food. Processing and storage are generally inevitable in cases where the ingredients spoil quickly and/or are rich in proteins. Storage raises the risk of accumulation of biogenic amines. It seems that their accumulation is totally dependent on the microorganisms that create histamine during food storage (especially in case of foods with a high L-histidine content) [35]. Overall, the fresher the food, the lower the probability of biogenic amine formation.

Amines are classified as monoamines, diamines and polyamines, depending on how many amine groups they contain. Among the most important biogenic amines found in food are monoamine tyramine, diamines histamine, putrescine and cadaverine, as well as the polyamines spermine and spermidine [36].

In the context of HIT syndrome, of clinical significance is tyramine, which may be present in excessive amounts in certain types of ripening cheeses. In sensitive people, this is related to increased blood pressure and the consequent occurrence of migraine pains [37].

Biogenic amines may contribute to histamine toxicity by saturating enzymes responsible for the degradation of histamine in the mucosa (DAO, HNMT). The diamines putrescine and cadaverine are considered to be the amines with the greatest influence on the metabolism of histamine. This is due to the fact that the DAO enzyme breaks down them preferentially [13,23,36]. Foods with a high biogenic amine content are generally considered as risky and should be omitted from low-histamine diets [9,23].

#### Values of Biogenic Amines and Histamine in Foods

**Figure 4:**
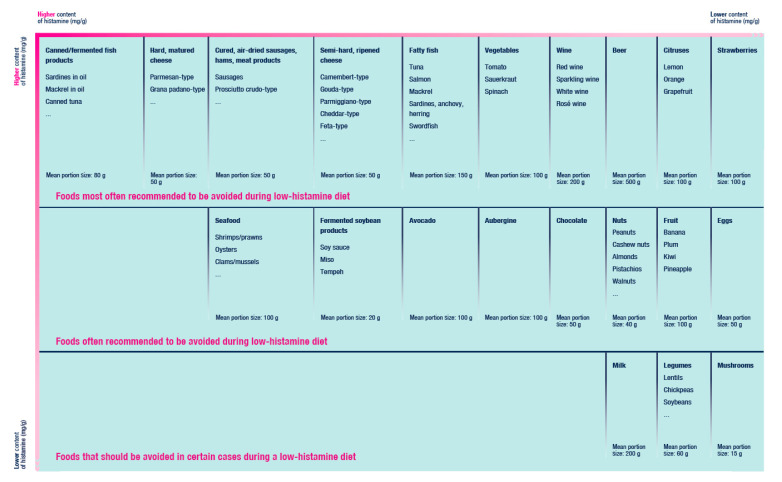
Foods that are most often recommended to be excluded from low-histamine diets. Adapted according to [9].

A diet that ensures the complete elimination of histamine is unattainable [38]. The content of biogenic amines and histamine in foods differs in dependence on their source, freshness, types, pH, salt content, content of proteins (and L-histidine), processing and storage [13,23,39]. The wide range of content of histamine and/or other biogenic amines for individual foods makes these parameters inconclusive and so we do not regard the listing of specific value intervals per 100 g of food as authoritative. In Figure 4, we present a list of foods that are most often recommended to be excluded from diet in case of suspected HIT.

In Table 3, we list foods that in usual quantities are considered safe from triggering HIT symptoms.

In general, biogenic amines are thermostable. If they are already present in the food, heat treatment does not significantly degrade them [36]. However, boiling in water can reduce the biogenic amines content in certain types of vegetables, most likely by transferring them from the food to the water. Boiling spinach reduced the histamine level by 83% when compared to raw spinach, while analysis confirmed the transfer of the histamine from the spinach to the water (Latorre-Moratalla et al., 2015, in [23]). Heat treatment needs not always lead to a reduction of the biogenic amines contained in the food however. Heat treatment in the form of boiling and grilling showed an increase of the biogenic amines content in mg/100 g in aubergine, green and yellow beans [41,42]. In a work by Chung, the histamine content in mg/100 g in grilled seafood and meat increased, whereas boiling these foods reduced the histamine content in the meat. Boiling the vegetable had no influence on the content of histamine or reduced it only minimally [43].

### 1.4. Factors Contributing to Increased Sensitivity to Histamine

Among the factors increasing the sensitivity of individuals to the ingestion of histamine are classed other biogenic amines, alcohol (blocks the enzyme DAO, can release endogenous histamine), specific medications (with an inhibitory effect on DAO) and malnutrition, leading to an insufficiency of enzyme cofactors (vitamin C, copper, vitamin B6) [13].

### 1.5. Microbiome and HIT

In 2018, Schink compared microbial patterns from 33 healthy individuals with 33 persons with suspected HIT, 8 of whom had decreased DAO enzyme activity in serum. In comparison with those patients suspected of HIT presence, the healthy people showed a greater abundance of the *Bifidobacteriaceae* family, with a median of 0.3% [44]. To this family belongs the *Bifidobacterium* genus, which confers health benefits to the host [45]. In persons having decreased DAO activity in serum, a greater abundance of the *Proteobacteria* genus was observed. The higher ratio in favour of *Proteobacteria* genus, which competes with strict anaerobes (including bacteria of the genus *Bifidobacterium*), may predict dysbiosis and/or impaired intestinal epithelial function [44]. If the bifidobacteria were used as a starting culture in the production of fermented sausages, the end products contained lower amounts of biogenic amines [46].

Some bacterial strains also have an enzyme that ensures endogenous histamine synthesis in the human body. It should be emphasized that the presence of bacterial L-histidine decarboxylase is strain-, not species-, specific [47]. Accordingly, it is not possible to extrapolate this property from one strain to another, although within the same species. Hence, it is imperative to always assess independently the amount of produced histamine for the individual strains of bacterial species.

Certain strains which are considered potentially probiotic, for example *Lactobacillus saerimneri* 30a, produce a significant amount of histamine and other biogenic amines [48,49,50]. In the wide commercially used bacteria *Limosilactobacillus reuteri* DSM 17938, the presence of genes responsible for the synthesis of this enzyme was not proven [51]. From the 15 strains of the bacteria *Lactobacillus acidophilus*, *Lacticaseibacillus casei*, *Lactobacillus delbrueckii* ssp. *bulgaricus*, *Lactobacillus lactis* ssp. *lactis*, *Lactococcus lactis* ssp. *lactis* and *Lactiplantibacillus plantarum*, only two strains, *L. casei* TISTR 389 and *L. bulgaricus* TISTR 895, appeared to be potentially histamine-producing [47].

Certain strains of the following bacteria, yeasts and moulds genera and species have the capacity for histamine formation. In Table 4, we summarize microbial genera and species in which the presence of gene for the L-histidine decarboxylase was demonstrated.

The presence of such bacteria in the gastrointestinal tract could for certain individuals increase sensitivity to ingested histamine. In a study from 2013, the effect of the histamine-producing strain of the *Lacticaseibacillus rhamnosus* species was studied in mice. Frei et al. came to the conclusion that alteration of the innate immune response (e.g., dendritic cells) may be mediated through the H_2_ receptor not only by endogenous histamine, but also by histamine produced by the microbiome [53]. The *L. rhamnosus* LGG and *L. rhamnosus* Lc705 strains suppressed the expression of the H_4_ receptor of mast cells and decreased mast cell activation and IgE response [54].

Since microorganisms play a crucial role in histamine formation; they have been studied for their ability to degrade biogenic amines in foods, particularly histamine and tyramine [55,56,57]. The *Lactiplantibacillus plantarum* D-103 strain was able to degrade histamine up to 100% in histamine MRS broth [57]. Although the microbial catabolic activities responsible for histamine degradation have yet to be completely elucidated, microbial copper-containing amine oxidases, such as histamine oxidase, are most likely involved [55,57]. The capacity of microorganisms to degrade biogenic amines is strain-specific [55]. In the future, eligible strains could be exploited to control the accumulation of biogenic amines in certain foods (e.g., cheese, wine, miso) [55,56,57]. This approach would require an excellent knowledge of the microbial metabolism, since some by-products of the enzymatic reactions (e.g., hydrogen peroxide) are not desirable [55]. Moreover, some strains could have properties for concomitant degradation and production of biogenic amines [56].

## 2. Diagnostic Approaches

HIT is currently not a nosological unit. Direct HIT-specific diagnostic criteria or markers are lacking [9]. Generally, for intolerances and malabsorption disorders, several diagnostic strategies using various tests have been proposed. In distinction to some other disorders such as for example celiac disease, there is no valid diagnostic laboratory assay for HIT that has been was consensually accepted [1,6,9,24].

It is proposed that the manifestation of HIT symptoms has its origin in a reduced level/activity of DAO. Based on this, the measurement of concentration or activity of this enzyme should be useful for the diagnosing of HIT. However, the problem resides in the fact that a reference value for DAO levels in serum has not yet been established [24,27]. Moreover, a measured DAO value and/or activity in a serum may be incoherent with the current level/functional activity of DAO in the intestinal mucosa.

### 2.1. DAO Enzyme Activity in Serum

The most studied but still controversial laboratory diagnostic approach is the analysis of DAO enzyme activity in serum. The tests measure the amount of histamine that degrades over a specified time in a blood sample, using enzyme-linked immunosorbent assay (ELISA) or radioimmunoassay (RIA). The threshold for serum DAO enzyme activity by which the presence of HIT should be considered has been proposed at 10 U/mL [58,59]. A number of authors have pointed out the potential contribution of this diagnostic approach in the search for the causes of the symptoms in patients with suspected HIT [9,59,60,61]. The reason for the controversy was that DAO was not detected in the blood by monoclonal antibodies, at least not in relevant amounts [62]. Accordingly, the DAO enzyme activity in serum could not be considered as conclusive [1]. In 2017, however, Boehm et al. demonstrated that ELISA is able to reliably and accurately quantify human DAO in different biological fluids and concluded that the potential of DAO as a biomarker in various diseases can be evaluated [27]. Still, in some patients, the relation between DAO activity in blood serum and the clinical history of symptomatic patients suffering from symptoms typical for HIT could not be confirmed. This can be explained by the fact that the activity of the DAO enzyme may naturally vary (over the course of the day and/or over the month) in an individual [63]. Measuring the DAO enzyme activity in serum may be considered as a part of the complementary examinations in certain cases if the determination of DAO activity in the intestinal mucosa is not feasible.

### 2.2. DAO Activity in the Intestinal Mucosa

Determination of DAO activity in the intestinal mucosa could represent a reliable diagnostic method. The determination is conditioned by a biopsy of colon tissue during colonoscopy. There are only a few studies addressing this diagnostic approach. However, they showed reduced DAO catabolic activity in patients with urticaria [64] and a food allergy [65] with associated higher histamine levels; the importance of this approach however needs to be confirmed by more robust data [1,9].

### 2.3. Faecal Histamine Levels

As was stated, the microbiota of the gastrointestinal tract can be a significant source of histamine [50]. The determination of faecal histamine levels is therefore not considered a reliable method [1].

### 2.4. Skin Prick Test

In HIT diagnostics the skin prick test variant, whose results are read after a longer time (50 min) than in the standard prick test (20 min), has been applied. The resolution of the redness from the puncture occurs later in symptomatic patients in comparison with the controls, which may signal a reduced ability of the body to degrade intracutaneously administered histamine. The limitation of this test resides in the fact that it need not necessarily reflect the degradation of histamine in the small intestine. Moreover, it is difficult, on the basis of this test, to differentiate HIT from other, for example allergic, disorders [66].

### 2.5. Histamine Levels in Plasma

Fourteen patients with suspected HIT were given orally 75 mg of histamine or a placebo, and plasmatic levels of histamine were measured at 10 min. intervals over 1 h. These results were compared with 4 healthy individuals. In this small sample, no significant differences were found in plasma histamine levels between the experimental and placebo groups. After the administration of the placebo, 33% of the patients had an increased level of histamine in the plasma, while about 50% exhibited symptoms [67].

### 2.6. The Histamine Challenge Test

The histamine challenge (provocation) test provides diagnostic output as well as a determination of the individual threshold dose of ingested histamine. The threshold dose of ingested histamine is the amount of histamine capable of triggering symptoms in an individual. On the other hand, it is difficult (or even impossible) to estimate the histamine content for each food because it varies depending on multiple factors [13,23,39]. The main disadvantage of this test is that it demands practically uninterrupted supervision by specialist personnel over a relatively long time-interval. In a number of the publications, the provocative dose was set at 75 mg of histamine [1], which should refer to a dose harmless to a healthy individual and a commonly occurring dietary dose [68]. However, a consensus on the critical value for histamine intoxication is lacking [10]. In a small placebo-controlled study, a dosage of 75 mg histamine immediately caused symptoms (tachycardia, sneezing, itchy nose, rhinorrhoea) in 5 of the 10 healthy patients tested [68]. An Austrian study from 2011 also questioned the decisiveness of the test. The patients were given 75 mg histamine, and those who reacted to it (n = 39), were divided into groups and given tea with/without a histamine and a capsule with a DAO enzyme, or a placebo. According to the study’s author, symptoms with patients suspected of HIT in individual organs manifested in a variety of situations. In some cases, the patients reacted unexpectedly, even randomly. For the diagnosing of HIT therefore, the reproducibility of the individual symptoms may not in itself be informative; a scoring system evaluating all symptoms could be more appropriate [16]. In addition, the histamine challenge (provocation) test itself carries the risk of serious side effects [9].

### 2.7. Single Nucleotide Polymorphisms (SNPs) of AOC1 Gene Evaluation

Currently, there are known 4 different SNPs of the AOC1 gene associated with a predisposition to HIT [21]. Genetic testing of AOC1 gene SNPs is a non-invasive procedure, where SNPs evaluated from blood or oral mucosa samples could be provided in ambulant care or at home. The results could be read in days. This simple test may play a part in the complementary testing of suspected individuals in order to support the results of other diagnostic outcomes.

### 2.8. Determination of Histamine and Its Metabolite 1-Methylhistamine from Urine

In 2017, Comas-Basté proposed a new diagnostic approach. The principle of this non-invasive test is the determination of histamine and its metabolite 1-methylhistamine from urine by ultra-high performance liquid chromatography and fluorimetry [69]. This approach however is waiting for validation [1,9].

**Figure 5:**
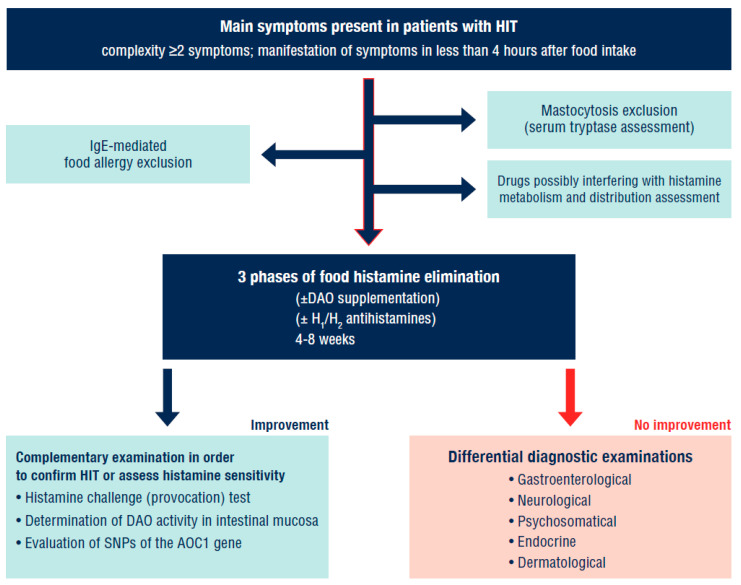
Diagnostic approach in a patient with suspected HIT. IgE—immunoglobulin E; DAO—diamine oxidase; HIT—histamine intolerance; SNPs—single nucleotide polymorphisms; AOC1—amine oxidase copper containing 1. Adapted according to [1,6,9].

As there is currently no validated diagnostic method which would unequivocally identify the intake of exogenous histamine as a trigger of symptoms, we would recommend the approach described in Figure 5. In future it may be optimal to add the determination of histamine and its metabolites in urine among the diagnostic procedures carried out before the actual elimination of food histamine.

Concerns have been raised regarding fact that HIT suspected patients often receive questionnaires from non-scientific sources and after filling them out they come to the specialist already convinced of their diagnosis and accordingly refuse further examination. Questionnaires from non-scientific sources are not considered to be a reliable diagnostic approach [70].

### 2.9. Differential Diagnostic Exclusion of Other Diseases

Symptoms triggered by the ingestion of histamine are non-specific. Within diagnostic approaches, the exclusion of allergic disorders is recommended as the first step (IgE-mediated food allergy). The excessive formation of histamine may also be related to rare, relatively well-differentiated specific disorders. For the exclusion of mastocytosis in HIT suspected patients, their serum tryptase levels should be examined [6]. The repeated taking of samples is optimal—during the course of the symptoms and then after their diminution. Chronic disorders (Crohn’s disease, celiac disease) should be excluded, along with a potential infectious noxa (*Helicobacter pylori*, zoonoses). During these first examinations, it is recommended for the patient to compile a patient’s diary, where the food consumed and symptoms manifested will be recorded. A comprehensible smartphone application developed in cooperation with specialists could be the option.

After the differential diagnostic exclusion of other possible causes of the symptoms, there follows a low-histamine diet, which should be divided into 3 phases (Table 5). When the effect of the diet is elusive, histamine exposure could follow (e.g., the consumption of sauerkraut). The onset of symptoms shortly after exposure and their subsequent remission after dieting resumption may indicate HIT.

If the response to a low-histamine diet is insufficient and no remission of symptoms has been observed, it is recommended to perform further professional examinations with specialists (gastroenterologist, neurologist, endocrinologist, dermatologist).

The HIT syndrome does not necessarily indicate one (transient) feature of the organism. Patients with inflammatory bowel diseases showed decreased serum DAO activity [13,71]. Similarly, in patients with ulcerative colitis, decreased DAO activity in the colonic mucosa has been shown [72]. The AOC1 gene polymorphism was related to the severity of the course of the ulcerative colitis [73]. The hypothesis that decreased DAO activity resulting from a gene polymorphism could act as a risk factor for the development of inflammatory bowel disease has not been proven [74]. It should be stated that with non-celiac gluten sensitivity have been reported intestinal and extra-intestinal symptoms which are very similar to those observed in HIT. Gluten-containing cereals are found in many foods, including bread, pasta, pizza, bulgur and couscous and in beverages such as beer. The majority of these foods and drinks however also contain histamine and/or are usually consumed with other products with a histamine content. Many bakery products containing gluten, and also beer, contain yeasts, thus live microorganisms which may contribute to the production of histamine are used in the process of their production. Bulgur, pasta and pizza are usually consumed with tomatoes and other vegetables which, due to their histamine content or ability to release endogenous histamine, could trigger symptoms in sensitive people [75].

In 18 of 20 patients with refractory celiac disease, Schnedl et al. diagnosed other intolerances/malabsorption and/or *H. pylori* infection. HIT syndrome was diagnosed in 11 patients. HIT would seem to play a significant role among patients with celiac disease who do not respond to therapeutic measures [76].

## 3. Therapeutic Approaches with HIT

Among therapeutic approaches, the gold standard is a low-histamine diet. A good response to such a diet is considered to be confirmation of HIT. Alongside the dietary measures, DAO supplementation supporting the degradation of ingested histamine is recommended as subsidiary treatment for individuals with intestinal DAO deficiency [9]. In acute and clinically more severe cases, or in cases where the ingested histamine cannot be completely removed by following the low-histamine diet [38], H_1_ (or possibly H_2_) antihistamines can be used (preferably short-term use).

### 3.1. Low-Histamine Diet

The principle of the low-histamine diet consists in a choice of foods where an excessive amount of histamine or biogenic amines respectively is not to be expected. In the first phase of the low-histamine diet, foods that typically contain a high amount of histamine (and other biogenic amines) should be completely excluded. There is a great variability between studies regarding type of foods that is recommended to avoid during elimination diet. Some of routinely excluded foods contain only low levels of biogenic amines and become designated as histamine liberators [77]. In Figure 4, we present examples of foods that are often recommended to exclude in low-histamine diet.

Foods that under normal circumstances do not contain high levels of biogenic amines should be consumed as fresh as possible (Table 3).

The low-histamine diet should be temporary and must not stress the patient. The patient should be aware that after certain period some eliminated foods may again be reintroduced to the menu.

Reese et al. proposed 3 phases during which the foods responsible for the symptoms are progressively eliminated and reintroduced respectively (Table 5). Adherence to a low-histamine diet has clearly led to improved gastrointestinal, cutaneous and neurological manifestations [23,78], and in some cases simultaneous raising of DAO serum levels was observed [79].

### 3.2. Exogenous Supply of DAO

Similarly as in lactose intolerance, lactase supplementation is used, the exogenous supply of the DAO enzyme has been proposed for HIT [9,16,32]. The European Food Safety Authority (EFSA) has authorized a porcine kidney extract containing 0.3 mg of DAO enzyme as a novel food. From 2002, it could be distributed on the market as a nutrition supplement and from 2013 as a food for special medical purposes. According to EFSA, the maximum daily dose of exogenously ingested enzyme is 3 × 0.3 mg, which corresponds to 0.9 mg of DAO. The dosage form must be gastro-resistant in order to deliver the enzyme undamaged to the place of presumed effect (small intestine) [80]. It has been demonstrated that DAO exogenous supplementation has improved the symptoms in clinical practice (Table 6).

Despite the promising results, studies dealing with DAO exogenous supplementation are scarce. In addition, the studies conducted investigated only a small sample of patients. It is therefore necessary to confirm the clinical significance of exogenous supplementation of the DAO enzyme by more robust, well-designed clinical trials.

#### Other Potential Sources of DAO

DAO need not be necessarily of animal origin. In 2020, Comas-Basté carried out screening of the ability of the family of the *Leguminosae* (legumes) plant to break down histamine. The goal was to identify plants with DAO enzyme activity (in vitro) and then to consider their potential usage as an active component of enzyme supplements. Lyophilization of the sprouts kept their enzyme activity unchanged for at least 12 months. The results show that in the future certain edible legumes could be suitable for the manufacture of supplements with DAO content for the management of HIT [83].

### 3.3. Antihistamines

The treatment of patients with antihistamines is empirical. There exist no randomized clinical trials that prove the contribution of this therapy in HIT. Therapeutic dosages and the choice of the generation and type of antihistamine (H_1_/H_2_) are in the competence of the clinician after the manifestation of the symptoms (gastrointestinal, neurological, dermatological) are taken into account; in consideration of effectiveness and safety however, 2nd or 3rd generation of H_1_ antihistamines should take precedence. H_2_ blockers could be used in patients with dominant gastrointestinal symptoms (hyperacidity and reflux as manifestations of HIT are also questions to discuss).

Treatment with antihistamines should be conscious and time-limited and should help create a picture of whether H_1_/H_2_ receptor blockade attenuates manifestations [1]. However, it may also be used as a therapeutic-diagnostic test.

### 3.4. Complementary Strategies in HIT Management

Some authors regard supplementation of cofactors of the DAO enzyme as optional adjunctive therapy. Vitamin C, copper or vitamin B6 supplementation may be considered [32,33,40,84].

Supplementation with probiotic microorganisms could lead to such modulations of microbiome that would reduce the production of the microbial enzyme L-histidine decarboxylase. The precondition therefore is the administration of strains that do not produce L-histidine decarboxylase. In an ideal case, these would be strains capable at the same time of degrading histamine (or other biogenic amines). Clinical trials assessing the possible impact of probiotic administration in HIT are not found in literature up to the present.

Arising from experimental trials, it would seem at present that members of *Bifidobacterium* genus could be considered as candidates for appropriate supplementation, but studies confirming this assumption are warranted [44,46].

## 4. Conclusions

HIT represents a set of diverse symptoms that appear following the ingestion of food with a content of such amounts of histamine that usually do not provoke symptoms in a healthy person. Manifestations may be caused by various pathophysiological mechanisms, or a combination of them. It would seem that symptoms typical for HIT in an individual may potentiate the presence of other disorders such as, e.g., celiac disease or *H. pylori* infection. The diagnosing of HIT therefore requires a complex time-demanding multidisciplinary approach, including the systematic elimination of disorders with a similar manifestation of symptoms. A low-histamine diet is currently a suitable (not however sole) diagnostic and at the same time therapeutic measure. Recently, evidence has been growing regarding the therapeutic contribution of food for special medical purposes containing a DAO enzyme of animal origin. H_1_/H_2_ antihistamines may also be considered in management of HIT, with the daily dosage (usually higher than the standard) determined by a clinician on the basis of the patient’s clinical condition. Pharmacological treatment should have a limited time duration. The aim of the therapeutic approach should be to develop such dietary habits which will not in the future lead to the triggering of symptoms.

## Figures and Tables

**Table 1:** Symptoms and differential diagnostics in patients with suspected adverse reactions to ingested histamine. Adapted according to Reese et al. 2017 [1].

Symptoms	Differential Diagnosis
Flushing	Neuroendocrine tumors
Itching	Urticaria, pruritus sine materia, prurigo
Nausea/vomiting/abdominal pain	Peptic ulcer disease, hiatal hernia, gastroesophageal reflux disease
Diarrhea and abdominal pain	Chronic inflammatory bowel disorders, disorders of carbohydrate metabolism
	(lactose intolerance, fructose malabsorption), celiac disease
Rhinitis	Allergic and non-allergic rhinitis of other origin
Dyspnea, dysphonia	Allergic and non-allergic asthma
Hypotension, vertigo, tachycardia	Anaphylaxis
Important differential diagnostic information is achieved by the analysis of symptoms with respect to their onset time. Adverse food reactions only considered if the symptoms manifested in less than 4 h from food intake.	

**Table 2:** Drugs and substances with possible effects on the metabolism and the distribution of histamine in the organism, which encompass decreasing DAO activity. Adapted according to [13,31,32,33,34].

**DAO blocking foods**	
Alcohol	wine and spirits especially
**OTC drugs interfering with DAO activity (decreasing DAO activity)**	
Expectorants, mucolytics	ambroxol, N-acetylcysteine
Nonsteroidal anti-inflammatory drugs	acetylsalicylic acid, ibuprofen
**Rx drugs interfering with histamine metabolism or distribution**	
Prokinetics	metoclopramide
Antiinfectives	clavulanic acid, isoniazid, cefuroxime, cefotiame, pentamidine, chloroquine, doxycycline, neomycin B, acriflavine, D-cycloserine
Bronchodilators	aminophylline, theophylline
Diuretics	amiloride, furosemide
Antidepressants	amitriptyline, monoaminooxidase 1 inhibitors
Anxiolytics	diazepam, barbiturates
Antipsychotics	haloperidol
Cytostatics	cyclophosphamide
Antihypertensives	verapamil, dihydrazine, alprenolol
Cardiotonics	dobutamine, dopamine
Opioids	pethidine, morphine, codeine
Analgesics	metamizole
Local anaesthetics	lidocaine, prilocaine, marcaine, procaine
General anaesthetics	thiopental
Muscle relaxants	pancuronium, alcuronium, D-tubocurarine
Antiarrhytmics	propafenone, verapamil, quinidine
**Antihistamines decreasing DAO activity**	
H_1_/H_2_ receptor blockers	cimetidine, promethazine
**Other substances interfering with histamine metabolism**	
Radiocontrast agents	iodine containing

OTC-over the counter.

**Table 3:** Foods that in usual quantities are considered safe from triggering HIT symptoms. Adapted according to [40].

Water, coffee, tea, homemade juices from allowed fruits and vegetables
Bread, pastry, potatoes, rice, pasta, cereals, millet, buckwheat, corn
Yoghurt, fresh soft cheese
Lettuce, cauliflower, broccoli, chicory, carrot, garlic, onion, cucumber, pumpkin, zucchini, pepper, radish, artichoke, rhubarb, asparagus
Apple, pear, cherry, amarelle, peach, apricot, watermelon, blueberries
Spices, herbs
Vegetable oil, vinegar
FRESH/IMMEDIATELY FROZEN meat: poultry, veal, beef, lamb, pork
FRESH/IMMEDIATELY FROZEN fish: cod/pollock, trout, zander, halibut
Ham (fresh, cooked and high-quality), eggs (cooked)
Jam made from allowed fruits, honey, butter, margarine

**Table 4:** Microbial genera or species which may have the capacity for histamine formation. Adapted according to [39,50,52,53].

Bacteria		
*Acinetobacter* spp.	*Chryseobacterium* spp.	*Pediococcus pentosaceus*
*Alcalingenes faecalis*	*Klebsiella oxytoca*	*Proteus* spp.
	*Klebsiella pneumoniae*	
*Arizona* spp.		*Providencia* spp.
	*Kluyvera* spp.	*Providencia heimbachae*
*Cedecea* spp.		
	*Lacticaseibacillus casei*	*Pseudomonas putida*
*Citrobacter freundii*	*Lacticaseibacillus paracasei*	*Pseudomonas lundensis*
*Citrobacter braakii*	*Lacticaseibacillus rhamnosus*	*Pseudomonas stutzeri*
	*Lactiplantibacillus plantarum*	
	*Lactobacillus curvatus*	
*Edwardsiella* spp.	*Lactobacillus delbrueckii*	*Psychrobacter* spp.
	*Lactobacillus helveticus*	
*Enterococcus casseliflavus*	*Lactococcus lactis* ssp. *lactis*	*Raoultella planticola*
*Enterococcus faecalis*	*Latilactobacillus* spp.	*Raoultella ornithinolytica*
*Enterococcus faecium*	*Lentilactobacillus buchneri*	
	*Lentilactobacillus hilgardii*	*Salmonella enterica* ssp. *arizonae*
*Enterobacter* spp.	*Lentilactobacillus parabuchneri*	
	*Leuconostoc* spp.	
*Escherichia coli*	*Levilactobacillus brevis*	*Serratia* spp.
*Escherichia fergusonii*	*Limosilactobacillus reuteri*	
	*Limosilactobacillus vaginalis*	*Sphingobacterium* spp.
*Hafnia alvei*		
*Hafnia paralvei*	*Microbacterium foliorum*	*Streptococcus thermophilus*
*Halomonas* spp.	*Morganella morganii*	*Tetragenococcus halophilus*
**Yeasts and moulds**		
*Debaryomyces hansenii*	*Geotrichum candidum*	

**Table 5:** Phases of dietary measures in patients with suspected HIT. Adapted according to [1].

Phase	Objective	Recommendation	Duration
Phase 1: Elimination phase	Reduction in symptoms to a maximum possible level	• Change in diet composition - introduction of mixed diet measures with accent on fresh vegetables and reduction of biogenic amine intake, in particular histamine • Nutrient optimization	10–14 days
Phase 2: Test phase	Reintroducing foods excluded in Phase 1, after taking into account individual risk factors (stress, menstruation, medication use etc.)	• Targeted gradual reintroduction of suspected foods taking into consideration patient’s individual dietary preferences • Assessment of individual sensitivity to ingested histamine	Up to 6 weeks
Phase 3: Long-term diet	Maintenance of high-quality of life Continual balanced diet	• Individual nutritional recommendations based on individual sensitivity to ingested histamine taking exogenous risk factors into consideration	_

**Table 6:** Summary of clinical studies testing the effects of exogenous supplementation of diamine oxidase obtained from porcine kidneys. DAO—diamine oxidase; DBPC—double-blind placebo controlled; N/A—not available; 5-HT—5 hydroxytryptamine; UAS7—Urticaria Activity Score 7; NS—non-significant; C1—before treatment (consultation no. 1); C2—after treatment (consultation no. 2) [16,58,59,81,82].

Study	Trial Design	Intervention	Control	Sample size	Duration of Intervention	Reported Outcomes
Komericki, 2011	DBPC cross-over study	0.5 mg DAO	Placebo	39	N/A	A significant improvement in symptoms after DAO administration as compared with placebo.
Manzotti, 2016	Restrospective observational study	2 × 0.3 mg DAO	N/A	14	14	13 patients (93%) reported improvement in ≥ 1 of symptoms.
Yacoub, 2018	DBPC cross-over study	2 × 0.3 mg DAO	Placebo	20	30	A significant improvement in Urticaria Activity Score 7 (UAS7) in patients with urticaria unsatisfactorily controlled by antihistamines (*p* < 0.05). Mild significant reduction in antihistamines consumption (*p* < 0.05).
Izquierdo-Casas, 2019	Randomized DBPC study	3 × 0.6 mg DAO	Placebo	82	C1 without an intervention (30 days) + C2 with intervention (30 days)	A significant reduction in number (*p* < 0.001) and duration (*p* < 0.05) of migraine episodes in intervention group compared with baseline. A decrease in the percentage of patients using selective 5-HT receptor agonists (triptans).
Schnedl, 2019	Open label interventional study	3 × 0.3 mg DAO	N/A	28	28 + 28 (follow-up)	Significant reduction in frequency and intensity of symptoms. 61% of patients showed mild increase in serum DAO levels (NS). During follow-up, without DAO supplementation, the symptoms sum score increased again and serum DAO levels slightly decreased.
